# State-of-the-art methods for quantifying microbial polyhydroxyalkanoates

**DOI:** 10.1128/aem.00274-25

**Published:** 2025-08-05

**Authors:** Eric M. Conners, Arpita Bose

**Affiliations:** 1 Department of Biology, Washington University in St. Louis 123752, St. Louis, Missouri, USA; Danmarks Tekniske Universitet The Novo Nordisk Foundation Center for Biosustainability, Kgs. Lyngby, Denmark

## Abstract

Polyhydroxyalkanoates are a diverse class of microbially synthesized polymers that are used to make bioplastics with a wide range of applications. As interest in polyhydroxyalkanoates (PHAs) grows, researchers are faced with a challenge: how best to use the resources at their disposal to reliably quantify PHA produced by their microbe(s) of choice. Investigators must weigh the pros and cons of each method against logistical constraints (e.g., time, money, and equipment) and technical concerns (e.g., accuracy and sensitivity). At the same time, the broader community of scientists researching PHAs should aspire to land on a set of best practices. To this end, we must continually audit our methods. Here, we offer readers a snapshot of popular and emerging approaches for quantifying PHA in the lab. For each method, we provide an overview**,** list the primary equipment, briefly describe the methods, including improvements or iterations, and discuss the pros and cons of the approach. Along the way, we highlight gaps in research and make recommendations about best practices and future directions.

## INTRODUCTION

Polyhydroxyalkanoates (PHAs) are a class of biodegradable polyesters synthesized by various microorganisms as intracellular carbon and energy storage compounds (1). PHAs can be grouped based on the number of carbon atoms within the underlying monomers (2). Short chain length (scl) PHAs have monomers with 3–5 carbon atoms and include polymers like poly(3-hydroxybutyrate) (PHB), the most well-studied PHA. Medium chain length (mcl) PHAs have monomers with 6–14 carbon atoms and include polymers like poly(3-hydroxyhexanoate). Finally, long-chain length PHAs have monomers with 14+ carbon atoms. PHAs can also exist as copolymers made up of distinct monomers. For instance, the copolymer poly(3-hydroxybutyrate-*co*-3-hydroxyhexanoate) [P(3HB-*co*-3HHx)] comprises the 3-hydroxybutyrate and 3-hydroxyhexanoate monomers (3). Due to their structural variation, biocompatibility, and potential to replace petroleum-based plastics, PHAs have garnered significant interest in environmental, biomedical, and industrial applications (1, 4, 5). Accurate and reliable quantification of PHAs is essential for optimizing production processes, understanding microbial physiology, and evaluating the efficiency of biotechnological applications. Over the years, a variety of analytical methods have been developed for PHA quantification, ranging from traditional gravimetric techniques (6) to chromatographic (7), spectroscopic (8), and fluorescent approaches (9).

Today, gas chromatography is the gold standard for quantifying intracellular PHA, owing to its reliability and accuracy (10, 11). However, it requires lengthy pretreatment protocols using harsh reagents, and in many cases, relies on large amounts of biomass (liters to tens of liters). These concerns can be addressed using solvent-free methods that reduce costs, minimize pretreatment time, and empower high-throughput quantification of low-biomass samples—all while maintaining accuracy (12, 13). Furthermore, broadening quantification methods beyond the conventional chromatographic approaches will empower labs that may not have access to the equipment or expertise required for conventional methods.

As we expand our toolkit, it is important to assess how new methods fare against established ones: do state-of-the-art approaches make established ones obsolete? Or does each method carry its own strengths and weaknesses that are suited for different situations? Authors such as Baidurah (14) recently tackled these questions by reviewing a wide range of quantitative and qualitative techniques for analyzing diverse biopolymers, though not exclusively PHA (14). Additionally, Cao et al. (9) critically reviewed fluorescent techniques for quantifying PHA (9). Our goal with this minireview is to focus exclusively on quantitative methods for assessing PHA that meet the following parameters: (i) provide quantitative data about total PHA content; quantitative data about individual monomers and qualitative data about monomer composition are optional but unnecessary; (ii) were published within the last 10 years, including iterations on established methods; and (iii) are applicable to laboratory scale research (e.g., cell cultures <50 mL and doable using commonly available laboratory equipment). Thus, this minireview covers two broad categories of methods: chromatographic methods (including gas chromatography-mass spectrometry, reactive pyrolysis-gas chromatography/mass spectrometry, high-performance liquid chromatography, and liquid chromatography-mass spectrometry) and spectroscopic methods (including Fourier transform infrared spectroscopy, nuclear magnetic resonance spectroscopy, and fluorescent quantification). Finally, we touch on the different “flavors” of PHA—namely, storage PHAs that bacteria use as carbon and energy reserves, oligomeric PHAs that play important physiological roles in diverse prokaryotes and eukaryotes, and short-chain PHAs that modify proteins via covalent bonding—and the tools researchers have used to analyze them. In doing so, we aim to provide a concise overview of PHA quantification methods that are relevant to researchers seeking to enter the field or looking to refine their approaches.

## EXTRACTING PHA

Because PHA is an intracellular biopolymer, it is often extracted before quantification, although there are exceptions. Thus, any discussion of PHA quantification must consider the extraction procedures. Abate et al. (15) recently reviewed polyhydroxyalkanoate extraction strategies in detail. Here, we will offer a brief overview to orient the reader with the options for extracting PHA.

PHA extraction can be solvent-based or solvent-free (Table 1) (15). The former makes use of solvents like dichloromethane, 1,2-dichloroethane, methylene chloride, or sodium hypochlorite to dissolve bacterial cells, with chloroform standing alone as the benchmark solvent for extracting PHA (12, 15). These solvents are PHA-friendly, providing high extraction yields and high polymer quality (12). However, most halogenated solvents are hazardous for both the environment and users (15). Furthermore, solvent-based approaches are costly due to the large amounts of solvent (up to 20× biomass) and anti-solvent (up to 10× volume of original PHA solution) for recovery (11, 12). These drawbacks have opened the door for sustainable and safe alternatives, including propylene carbonate (16), dimethyl carbonate (17), supercritical fluids (18), ionic liquids (19), bio-based green solvents, and natural deep eutectic solvents (12).

**TABLE 1:** Methods for extracting polyhydroxyalkanoates from microbial cells

Method	Pros	Cons	Source
Solvent based			
Dichloromethane	Widely adopted, ease of use, and high recovery efficiency	Probable carcinogen and environmental toxin	(12, 15)
1,2-Dichloroethane			
Chloroform			
Dimethyl carbonate	Provisionally non-hazardous	Few lab-scale studies	(15, 20)
Solvent free			
Mechanical (e.g., high-pressure homogenization)	Additive-free	Requires high-pressure homogenizer	(13)
Detergents (e.g., SDS)	Effective at high cell densities with minimal impact on PHA yield and purity	Not economically competitive	(21)
Alkaline (e.g., NaOH)	High recovery yields and purity	May reduce polymer mass	(22)
Enzymatic	High recovery yields and purity	High cost	(23)

Alternatively, solvent-free methods relying on enzymes, surfactants, oxidants, alkali reagents, and mechanical disruption can be used to disrupt cells and release PHA (12, 13). However, they are less effective than solvents in terms of PHA recovery yields, purity, and economic cost (12). Thus, while solvent- or additive-free extraction methods are attractive from a health and safety perspective, more work is needed to make them competitive with traditional extraction methods.

Given the variety of extraction approaches available, one might be tempted to “mix and match” their preferred extraction and quantification procedures. However, it is important to recognize that pretreatment can significantly impact downstream quantification steps. For instance, extraction processes can affect the characteristics of PHA, including its crystallinity, molecular weight, purity, and PHA yield (24). Additionally, not all pretreatment steps are compatible with every quantification method. An in-depth discussion of extraction/quantification pairings is beyond the scope of this minireview. Future literature reviews or experimental work can examine how different pairings fare in the lab. Regardless, any new pairing of PHA extraction and quantification methods must be validated in the lab.

## CHROMATOGRAPHIC METHODS FOR QUANTIFYING PHA

The first chromatographic method for determining PHA content was published in 1978 by Braunegg et al. Using a solvent-based extraction protocol, they isolated poly-β-hydroxybutyric acid (PHB) from the Gram-negative bacterium *Alcaligenes eutrophus* (now known as *Cupriavidus necator*)*,* followed by quantification using a gas chromatograph equipped with a flame ionization detector. This process took 4 hours from sampling to quantification, with detection as low as 10^−5^ g PHB/L (10).

This basic framework—wherein PHA is released from cells and quantified via gas chromatography—remains the most common workflow used to measure PHA (11). But it has also been expanded and iterated upon. Below, we will discuss four chromatographic methods: gas chromatography-mass spectrometry (GC-MS), reactive pyrolysis GC-MS (Py-GC/MS), high-performance liquid chromatography (HPLC), and liquid chromatography-mass spectrometry (LC-MS).

### Gas chromatography-mass spectrometry

#### Overview

Gas chromatography (GC) is an analytical separation technique that provides both quantitative and qualitative information about a complex chemical mixture. During GC, volatile compounds are vaporized and carried along a column by the continuous flow of an inert gas (e.g., helium). The vaporized constituents of the original sample are separated based on their chemical and physical properties, at which point a detector (e.g., a flame ionization detector or barrier discharge ionization detector) identifies the constituent parts. This is visualized as a chromatogram with characteristic peaks corresponding to each constituent. The area under each peak is proportional to the amount in the original sample (14). By measuring internal standards (e.g., commercially available PHA polymers) alongside their samples, operators can generate a standard curve that is used to calculate the amount of PHA in each sample. When appropriate commercial standards are analyzed via GC coupled to mass spectrometry (GC-MS) and compared against a comprehensive database such as the National Institute of Standards and Technology, researchers can glean qualitative information on the identity of specific PHA monomers in samples where the biopolymer composition is unknown (25).

#### Method

The original GC method for PHA quantification, established in 1978, is still the basis for GC methods today (10). It involves four basic steps:

- Cell lysis: cells are suspended in an acidic methanol solution with H_2_SO_4_ and chloroform. The strength of the acid solution and duration of the incubation depend on the polymer type (e.g., scl or mcl PHA) and biomass and should be optimized accordingly.
- PHA depolymerization and transmethylation of resulting PHA monomers: this requires heating the cell suspension to approximately 100°C for one to several hours and converts extracted PHA to methyl esters. As with cell lysis, this step needs to be optimized (e.g., time and temperature) for the polymer and biomass in question.
- Separation of transmethylated PHA monomers: a liquid-liquid phase separation relying on vacuum centrifugation.
- Measurement of extracted methyl esters via GC and analysis with backend software.

#### Equipment and logistics

A GC instrument equipped with a column and detector is used to measure volatile methyl esters produced from PHA monomers during the pretreatment steps. Columns should be selected based on four parameters:

- Stationary phase: the polarity of the stationary phase should match the polarity of the analyte(s). PHA monomers may be polar or non-polar, though non-polar columns are commonly used.
- Column dimensions: longer columns provide higher resolution at the expense of increased run time; smaller internal diameters restrict sample capacity; thicker films work well at high sample concentrations and less volatile analytes.
- Temperature range: the column’s maximum temperature must exceed the highest temperature required for analysis; for PHAs, maximum column temperature is typically around 220°C–320°C (26–29).
- Column compatibility: operators should verify with manufacturers that a column works with their GC instrument and detector.

Commonly used columns for measuring PHA include the HP-5 (Agilent Technologies) (28), DB-WAX (Agilent Technologies) (27), SPB-1 capillary column (Supelco) (30), and Rxi-5 ms (Restek) (31), or Rt-S-BOND PLOT Column (Restek, USA) (32, 33). These columns are typically paired with flame ionization detectors (FID) for sensitive detection of PHA monomers. FIDs are two to three times more sensitive than thermal conductivity detectors and are highly sensitive to hydrocarbons (down to 10^−12^ g), making them well-suited for detecting PHA (34). FIDs are also low-cost and simpler to maintain, requiring only hydrogen and air. Alternatively, barrier ionization detectors (BIDs)—which use a plasma discharge (typically helium) to ionize analytes—are substantially more sensitive to a broader range of organic and inorganic compounds. This makes them costlier and slightly more onerous to operate and maintain. Thus, FID is likely ideal if PHA quantification is the sole goal, whereas BID offers flexibility toward other applications.

GC instruments can also be interfaced with an autosampler to increase throughput, which is especially useful for labs eager to screen large numbers of samples (e.g., when assessing PHA production across numerous strains or growth conditions). Pairing with mass spectrometry (GC-MS) allows researchers to determine the relative amounts of PHA monomers when the underlying monomer composition is unknown (35). Finally, operators must use commercially available standards to generate calibration curves. Ideally, these standards should mirror the biopolymers produced by the microbial chassis being studied.

GC is among the more labor- and time-intensive procedures. Altogether, this workflow takes >30 hours, depending on the number of samples being assessed (9). Additionally, it is vital for researchers to consider variation in sample processing times between pure polymers used as internal controls and different types of biomass. For example, methanolysis is more efficient in pure polymer compared to biomass. Researchers must conduct preliminary experiments to determine whether a correction between biomass and pure polymer is necessary. Assuming all equipment and reagents are on hand and all work is done in-house, most labs should expect 1–2 days for extraction, processing, and data analysis.

#### Pros and cons

Today, GC-FID is the gold standard approach (36). Thus, anyone seeking to break into the field will be flush with resources and will have an easier time comparing data across studies. However, sample preparation is labor-intensive and relies on hazardous chemicals (although it is simpler and cheaper compared to nuclear magnetic resonance [NMR] spectroscopy) (29). It is also not as amenable to high-throughput processing as other methods, particularly fluorescent (9).

### Reactive pyrolysis-GC/MS

#### Overview

Reactive Py-GC/MS mitigates the elaborate pretreatment processes that plague conventional GC methods (37). Originally developed in 1989 to characterize synthetic and natural polymers with ester bonds and/or polar groups, Py-GC/MS has become a powerful tool for identifying and quantifying microbial PHA (37, 38). Py-GC/MS is a thermal destructive analytical method that combines thermal decomposition (pyrolysis) with chemical derivatization to measure volatile and non-volatile samples alike (37). It does not require extensive pretreatment—dried biomass can be added directly into the Py-GC/MS apparatus—making it faster and less resource-intensive than conventional GC. Furthermore, PHA measures by Py-GC/MS highly correlate with those recorded by conventional GC, with R^2^ >0.9766 across multiple independent studies (39, 40). Thus, Py-GC/MS is a time-saving iteration on the gold standard method for PHA quantification.

#### Method

Unlike conventional GC methods, microbial cells can be added directly into the Py-GC/MS workflow, e.g., as a dried powder (39). The amount of sample mass is minuscule (typically 10–100 µg of dried cells), with numerous studies reporting inputs as low as 30 ug of pure polymer (41) or 1 mg dry cell biomass (39). In one study, only 100 µg of dried *C. necator* cells were needed to detect PHB as low as ~15 wt% (40). The samples are mixed with an organic alkali—typically tetramethyl ammonium hydroxide (TMAH)—that acts as both a hydrolysis and methylating agent (14). The cell/TMAH suspension is mixed in a small platinum cup that is dropped directly into a microfurnace pyrolyzer, which is then rapidly heated to 400°C–600°C to break down large molecules into smaller, more volatile fragments (14, 37). Following pyrolysis, the volatile derivatives are introduced into a GC, where they are separated into their constituent parts as per a typical GC. When paired with MS, the system can produce characteristic mass spectra for identification and quantification.

#### Equipment and logistics

Py-GC/MS takes advantage of a microfurnace pyrolyzer attached to a GC equipped with a detector (e.g., FID or BID) (14). Microfurnace pyrolyzers include single-shot (e.g., PY-3030S, Frontier Labs, USA) and multi-shot (e.g., EGA/PY-3030D, Frontier Labs, USA) models, with the latter offering more methodological flexibility at the expense of higher cost and greater complexity. Multi-shot pyrolyzers can perform multiple analytical modes (e.g., evolved gas analysis, reactive pyrolysis, single-shot pyrolysis, multi-shot pyrolysis, and thermal desorption), which allows labs to measure a wide range of compounds beyond PHA (37, 41). Most microfurnace pyrolyzers can be directly interfaced with GC injectors, making it an attractive option for labs already equipped with GC systems.

Another key component to Py-GC/MS is the organic alkali reagent, the most popular of which is TMAH. TMAH has several advantages over other organic alkali reagents: it is a highly efficient hydrolysis and methylating reagent, and it decomposes into tetramethylamine and methanol at high temperatures, which does not damage the column (14).

Assuming the lab is equipped with a GC, the microfurnace pyrolyzer is the primary upfront capital expenditure. However, it brings several benefits that can improve overall productivity: the abbreviated pretreatment steps use fewer consumables and take less time than conventional methods, which lowers costs and frees researchers to complete other tasks. Otherwise, data collection and analysis are similar to conventional methods, making it easy to compare data across studies and integrate into existing workflows.

#### Pros and cons

Py-GC/MS achieves sensitive, direct, and fast measurement of intracellular PHA without the labor- or resource-intensive pretreatment steps typically used for GC, while being just as reliable as conventional GC approaches (39, 40). Additionally, the biomass requirements are minimal, ranging from 10 to 100 µg of dried cells. This makes Py-GC/MS a time-saving and accurate iteration on conventional methods, though investigators must purchase specialized equipment that interfaces with their existing GC instruments.

There are also unresolved questions about the accuracy of Py-GC relative to conventional GC. Two independent comparisons between the two showed that Py-GC estimated greater intracellular PHA content than conventional GC in *C. necator* and *Bacillus* sp. The authors attributed this to the higher reaction efficiency of the intracellular biopolymer with TMAH (39, 40). This suggests that conventional methods underestimate actual PHA content, and future research should explore this possibility. Specifically, researchers interested in developing a Py-GC protocol should first conduct a complete PHA extraction and validate it to ensure it approaches 100%. The extracted PHA can then be quantified using a conventional GC approach alongside a Py-GC protocol, and the results correlated between the two methods. Commercial standards of known composition and quantity should be processed in parallel for calibration.

### High-performance liquid chromatography

#### Overview

In 1983, Karr et al. quantified PHB produced by *Rhizobium japonicum* using HPLC coupled with UV detection. Their approach, which strongly agreed with quantification by GC (R^2^ = 0.980), rapidly measured samples with as little as 0.01 µg PHB, a significant improvement over contemporaneous methods (7). To this day, HPLC provides both quantitative and qualitative information on PHAs, especially when researchers need to determine the monomeric composition of copolymers (42).

#### Method

Satoh et al. describe an HPLC approach beginning with an alkali-based PHA extraction (43). Here, 1 mL of activated sludge is digested in NaOH at 105°C, cooled to room temperature, and mixed with 2 N H_2_SO_4_ to form 2-butenoate (2BE) and 2-pentoenoate derivatives (2PE). These were then analyzed by HPLC on a Hitachi 2100 LaChromeElite with UV detection at 210 nm equipped with a SCR-101H column. Standard solutions of 2BE and 2PE were prepared and measured in parallel to generate a standard curve for calculating the amount of 2BE and 2PE in the sample extracts. From here, the authors determined the biopolymer concentrations (3-hydroxybutyrate [3HB] and 3-hydroxyvalerate [3HV]) in the original samples based on the conversion yields of 3HB and 3HV into the 2BE and 2PE derivatives. This workflow was later used to quantify PHA monomer fractions in *C. necator* and exhibited strong agreement with conventional GC (R^2^ > 0.9946) (42). More recently, HPLC achieved detection limits as low as 0.30 g PHA/L—and in the case of pure standards, detection was achievable as low as 0.020 g/L (44).

Thiele et al. adapted a novel HPLC approach that took advantage of high-pressure homogenization to disrupt cells and extract PHA. In brief, a high-pressure laboratory homogenizer (NS1001L2K, Niro Soavi, Italy) applied three passes of 600–1,200 bar of pressure to disrupt *C. necator* cells and release intracellular PHA. Using this approach, the authors achieved reproducibly high total PHA yields (>88%) and purities (>96%) with minimal washing. From here, they quantified the homopolymer poly(3-hydroxybutyrate [P(3HB)] and the copolymer poly(3-hydroxybutyrate-*co*-3-hydroxyhexanoate) [P(3HB-*co*-3HHx)] using an HPLC equipped with a diode array detector (HPLC-DAD) (Agilent 1200 series DAD, Agilent Technologies, USA). Their method resulted in near 100% P(3HB) and P(3HB-*co*-3HHx) yields at purities of 85%–100% (13).

#### Equipment and logistics

Many of the same principles that form GC apply to HPLC. At the heart of this HPLC system is the column, which contains the stationary phase that separates the sample into its constituent parts. Longer columns enable better resolution but increase runtime. Columns are interfaced with detectors that identify and quantify the constituents as they elute from the column. Common detectors include UV-Vis, fluorescence detectors, refractive index detectors, and mass spectrometers (i.e., LC-MS, discussed below). As with GC, autosamplers increase processing speed while maintaining consistency, which is vital for labs seeking high-throughput quantification. An example HPLC setup for quantifying PHA is as follows: Waters Alliance 2695 separation module (Waters, Milford, MA, USA) equipped with Rezex ROA-organic acid H+ (8%) (Phenomenex, 300 mm × 7.8 mm) coupled to a Waters 2996 UV detector set at 240 nm (44).

Ciliberti et al. established an HPLC protocol that took approximately 40 minutes per sample (44). PHA extraction adds an additional 1–2 hours, though this can vary depending on the extraction protocol.

#### Pros and cons

HPLC can analyze a broad range of non-volatile, polar, and thermally labile compounds. This makes it ideal not just for quantifying PHA, but other substances relevant to questions about microbial metabolism (e.g., nitrogenous compounds and organics) (45). Furthermore, HPLC avoids the time-consuming and energy-intensive step of lyophilizing or otherwise drying biomass, which is necessary for some approaches (e.g., GC). However, HPLC separations generally take longer than GC, use larger volumes of expensive consumables, and require specialized maintenance, thus increasing operational costs. It may not be an attractive approach for users seeking a simplified routine analysis pipeline, but for those seeking a more robust tool with broader applications, it may be worth the investment.

### Liquid chromatography-mass spectrometry

#### Overview

LC-MS combines the physical separation of HPLC with the mass analysis of mass spectrometry to provide quantitative and qualitative information about a wide range of compounds, including small molecules, peptides, proteins, lipids, and polymers. Much like GC-MS, LC-MS measures the amount of each compound present in a sample by separating it into its component parts and measuring the corresponding peak areas along a chromatogram. Peak areas are converted to mass values based on internal standards.

LC-MS is a fairly uncommon PHA quantification method. It has recently been applied to measure PHA produced by a handful of haloarchaea and bacteria (32, 33, 46–48). Though uncommon, it offers a viable route for quantifying PHA to those with access to LC instruments. Furthermore, it may prove to be more sensitive than conventional GC approaches.

#### Method

Because LC-MS is a relatively uncommon approach, methods have only been refined across a handful of studies. Solvent-based extraction methods are often used to convert intracellular PHA to derivatives that are measured by the LC-MS system. Hagagy et al. extracted PHA from 100 mL of *Halolamina* spp. cell culture by incubating dried cell pellets in 6% (wt/vol) sodium hypochlorite (37°C for 10 minutes), washing with acetone and ethanol (30:70), and dissolving pellets in hot chloroform. Finally, dissolved cells were filtered, and the filtrate was evaporated in a 40°C oven, leaving the extracted polymer, which was quantified via LC-MS. PHA quantities were normalized to cell dry weight, which was measured gravimetrically (47).

A slightly different LC-MS approach was used to quantify PHA produced by various purple non-sulfur bacteria (32, 33, 48). Here, the authors converted intracellular PHA to crotonic acid, which was then quantified via LC-MS. In brief, 10 mL of cell cultures were pelleted and suspended in a methanol/chloroform/sulfuric acid solution, followed by a 1 hour digestion at 95°C. Samples then underwent liquid-liquid phase separation via centrifugation to separate the organic and aqueous phases, followed by an overnight vacuum concentration to evaporate the aqueous phase, leaving behind the crotonic acid-containing organic phase. This was resuspended in 1:1 acetonitrile:water and filtered for LC-MS analysis, with crotonic acid being detected at a mass-to-charge ratio (*m/z*) of 87. Importantly, commercially available PHA was processed in parallel, acting as an internal standard that was essential for calculating the amount of PHA in each unknown sample. PHA quantities were normalized across various biomass parameters, including optical density, total protein, cell dry weight, and cell fresh weight.

#### Equipment and logistics

Many of the same equipment requirements applicable to GC-MS apply to LC-MS. In addition to an LC instrument (e.g., Agilent Technologies 6420 Triple Quad LC-MS), operators must select an appropriate column (e.g., Hypercarb or Hypersii ODS columns [Thermo Fisher Scientific, USA]) (33, 47). Detection is conducted by the mass spectrometer. There are three types of MS pertinent to PHA detection: electrospray single quadrupole (SQ), triple quadrupole (TQ), or quadrupole time of flight (Q-TOF), with sensitivity and resolution increasing from SQ to TQ to Q-TOF (49). Finally, downstream data analysis software (e.g., Agilent MassHunter, Agilent Technologies, USA) is used to quantify PHA monomers based on the resulting chromatograms.

Time to completion largely depends upon the extraction method, the LC-MS protocol, and the number of samples. Considering the extensive pretreatment steps and longer runtime for LC-MS compared to GC-MS, it is reasonable to assume that an LC-MS approach will be about as long or longer than a GC-MS approach (about 1–2 days). As with HPLC, LC-MS systems are generally more costly and complicated to operate than GC-MS systems, making them a less attractive option for inexperienced users. Additionally, the slightly more complicated sample pretreatment requirements can increase costs in terms of reagents, consumables, and operator time.

#### Pros and cons

One potential advantage of LC-MS over other chromatographic methods is its sensitivity: Conners et al. show that a conventional GC approach could not detect a commercially available PHB standard below 100 ppm, whereas their LC approach achieved detection as low as 1 ppm (32). Likewise, their LC method detected PHA extracted from bacterial cells, whereas the GC method could not, presumably due to small amounts of biomass used for extraction. Thus, an LC approach could open the door for small volume (<15 mL) growth experiments, which would make it easier to screen large numbers of strains and growth conditions while maintaining accuracy. However, a more comprehensive analysis that explores alternative extraction protocols, quantification parameters, and microbial chassis is needed to shed light on the detection limits of each approach.

## SPECTROSCOPIC METHODS FOR QUANTIFYING PHA

There are numerous spectroscopic methods for analyzing PHA. These include Fourier transform infrared (FTIR) spectroscopy, NMR spectroscopy, and fluorescence-based methods (9). These methods vary in their usefulness as purely quantitative methods. However, they are gaining popularity as simpler alternatives to the more labor-intensive methods above and are thus worthy of inspection.

### Fourier transform infrared spectroscopy

#### Overview

FTIR was initially established as a rapid method for detecting PHA in intact cells (50). Over the years, it has been deployed as a qualitative or semi-quantitative method, often relying on GC to quantify standards as the reference material and/or complex multivariate statistics or chemometrics for quantification (51–53). Recent work outlined below has explored the possibility of using FTIR as a fully independent method for quantifying PHA. Given FTIR’s popularity as a qualitative, semi-quantitative, and perhaps fully quantitative tool, it warrants further discussion here.

#### Method

When exposed to infrared light, molecules emit characteristic vibrational frequencies according to the underlying chemical bonds and functional groups. FTIR exploits this principle to generate unique “fingerprint” spectra of diverse samples—including solids, liquids, and gases—that reveal their underlying molecular structure. When applied to intact cells, the resulting spectra reflect that cell population’s total biochemical composition (8). This includes intracellular PHA, which shows a strong ester carbonyl group C=O band between 1,728 cm^−1^ and 1,744 cm^−1^ (51). For quantification, FTIR spectra historically needed to be calibrated with a known reference, often via GC or HPLC (51, 53, 54). This led to the development of mathematical models such as multivariate statistics or chemometrics for determining analyte concentrations from spectra (51, 53). In 2010, Arcos-Hernandez et al. (55) proposed the following workflow for quantifying PHA with FTIR:

- Samples are prepared by pelleting and washing 1 mL of cells. Then, a thin layer of wet solid biomass is smeared on a microscopic glass and dried.
- FTIR spectra are acquired using an FTIR spectrometer. An attenuated total reflection (ATR) technique is preferred because of its strong mid-infrared absorption of water while ensuring a short optical path for measurement (54). Three to ten replicates are recorded per sample.
- Data undergo preprocessing and analysis.
- A calibration model is generated via the partial least squares method.
- In parallel, reference data for model calibration are prepared following a standard GC workflow for PHA quantification.

In theory, the calibration model and reference analyses should only need to be conducted once per system. This means that new calibrations and references are needed for each new experimental setup (e.g., changing microbes or growth conditions).

Deng et al. recently explored FTIR as an independent method for quantifying PHA in 14 wastewater sludge samples (51). However, their method consistently underestimated actual PHA content (as measured by GC) by 47%–94%, which the authors attribute to extraction efficiency and the lack of proper internal standards. Thus, it remains unclear whether FTIR alone can provide accurate quantitative information. Additional studies are needed to explore whether improved extraction methods, alternative internal standards, or advanced computational tools can make FTIR a reliable standalone tool.

#### Equipment and logistics

The key piece of equipment is an FTIR spectrometer (e.g., an Agilent Cary 670 FTIR, Agilent Technologies, USA). To take advantage of the preferred ATR technique, spectrometers must be outfitted with an accessory that houses the ATR crystal (e.g., diamond, germanium, zinc selenide, or potassium bromide) and facilitates sample interaction (56). Until methods for using FTIR as a standalone quantitative tool are validated, researchers must also have access to GC, HPLC, or another “conventional” tool for calibrating standards.

Analytical time for quantification in mixed microbial cultures was reduced to under 30 minutes while maintaining comparable accuracy and reproducibility to GC (55). However, the added time of establishing standards and mathematical models makes quantification with FTIR potentially time-consuming up front. Once standards and models are developed and an FTIR method is validated for a given system, FTIR is theoretically a time-saving process compared to conventional chromatographic approaches.

#### Pros and cons

Pros of FTIR include the small sample size requirement (0.4 mg of biomass), minimal sample preparation, and rapid analysis time. However, it still depends on GC analysis to quantify the standard samples as the reference material (51, 55). Furthermore, FTIR requires multivariate statistics or chemometrics for calibration, which introduces additional complexity to data analysis (51, 53).

### Nuclear magnetic resonance spectroscopy

#### Overview

PHA quantification using NMR spectroscopy was first demonstrated in 1986 (57). Since then, it has been used to acquire both quantitative and qualitative information about microbial PHA. This is because PHAs exhibit characteristic NMR signals that can be assessed via ^1^H and ^13^C NMR analyses, the former of which is useful for quantification while the latter provides information on monomer composition (58).

#### Method

Following PHA extraction (typically solvent-based), samples are dissolved in a labeled solvent (e.g., deuterated chloroform) and run on an NMR spectrometer (e.g., Bruker Advance III NMR spectrometer, Bruker, USA, or Jeol ECX-300 spectrometer, Jeol, USA) (59, 60). Recently, Thiele et al. developed an on-line (i.e., integrated into a benchtop workflow for real-time processing without requiring separate sample collection or processing) NMR method using a benchtop NMR spectrometer (Spinsolve 43 Carbon Ultra NMR spectrometer, Magritek GmbH, Germany), wherein the spectrometer was connected directly to a batch reactor via a polytetrafluoroethylene tube connected to a syringe pump (58). This allowed them to transfer samples directly from the extraction vessel to the benchtop NMR by suction. By controlling the pump and spectrometer using a PLC controller, the authors achieved real-time on-line quantification of PHA copolymers during a 38 second measurement cycle. Importantly, their approach strongly agreed with a conventional GC method (R^2^ > 0.94).

#### Equipment and logistics

Researchers need access to labeled solvents (e.g., deuterated chloroform) and an NMR spectrometer (e.g., Bruker, JEOL, Agilent). As with most approaches, internal standards (e.g., commercially available PHA) are essential for calibration. Finally, analysis software (e.g., TopSpin, Bruker, USA) is required for downstream quantification of raw data.

#### Pros and cons

NMR is non-destructive, highly accurate, and reproducible and provides both quantitative and qualitative information. A key advantage of NMR is its ability to identify monomers and complex copolymers that have not been previously described (61). Yet, a typical NMR approach may be unsuitable for routine analysis due to the relatively large sample size requirements (tens of milligrams) (14). Additionally, PHA’s low solubility in solvents makes NMR characterization cumbersome (14). However, the on-line approach described by Thiele et al. may offer a more practical method, though it requires investment in a benchtop NMR device (58).

### Fluorescent quantification

#### Overview

Fluorescent methods for quantifying PHA rely on the relationship between the fluorescence response of stained PHA and PHA concentrations (62). Cao et al. (9) present an in-depth review of a wide range of fluorescent quantification (PHA-FQ) methods. Below, we briefly highlight the most relevant quantitative methods along with recent developments.

#### Method

PHA-FQ methods generally use five primary fluorochromes: Nile Blue A, Nile Red, LipidGreen1, LipidGreen2, and BODIPY (9). A typical PHA-FQ workflow requires investigators to grow cells, dissolve cells in an appropriate solvent dictated by the fluorochrome(s)—commonly acetone, DMSO, ethanol, or DMF—mix dissolved cells with the fluorochrome(s), incubate at a given temperature for a given length of time, and then measure fluorescence intensity using a spectrofluorometer or flow cytometer (9). Optimized procedures can take as little as 35 minutes, though shorter times are possible when automated pipelines are in place (63–65).

One challenge posed by fluorescence-based methods lies in the problem of delayed measurement—usually due to washing, staining, and other preparatory steps—which precludes real-time information about the state of intracellular PHA (66). Recent innovations have taken advantage of computational tools to develop rapid and on-line methods of PHA quantification based on fluorescence data. For instance, Sahu et al. developed a partial least squares (PLS) regression model to track PHA accumulation in real-time (67). Here, PLS based on Sudan black adsorption data, culture optical density, and intracellular protein measurements achieved a limit of detection of 0.036 g/L, with a significant reduction in processing time (190 minutes) compared to a traditional protocol (940 minutes). Importantly, these data were generated from only 10 mL of *C. necator* cell culture, negating the need for multi-liter cell cultures. Future work taking advantage of machine learning tools may empower researchers to use fluorescent signals as reliable markers for intracellular PHA, especially if new fluorochromes with better specificity and stability are developed (9).

Others have combined computational tools with two-dimensional (2D) fluorescence spectroscopy to quantify PHA in real time using fluorescent signals. 2D fluorescence spectroscopy scans several excitation and emission wavelengths simultaneously to generate excitation-emission matrices (EEMs) that record the intensity of each Ex/Em pair (68). This allows for non-destructive, rapid, *in situ* data collection. Recently, 2D fluorescence spectroscopy was used to generate EEMs from a 2 L bioreactor containing activated sludge from a wastewater treatment plant (66). The authors then compressed the data recorded in the EEMs via principal component analysis; the resulting principal components were used as inputs for mathematical correlational models obtained using “projection to latent structures modeling” to predict intracellular PHA content, with R^2^ = 0.76 for their training data set and an average error of 4.0% g_PHA_/ g_TS_, where g_TS_ refers to the solids weight after lyophilization. However, the authors point out that 2D fluorescence spectroscopy is heavily influenced by the specific culture conditions; media, biomass, and other culture constituents will influence the fluorescent signals and therefore the resulting EEMs. Thus, each new application of 2D fluorescence spectroscopy coupled with computational modeling must be calibrated to the system being measured. Future work that applies these tools in novel contexts will help researchers better understand when and how to apply them. Regardless, the method described by Guarda et al. shows promise as a rapid on-line method for quantifying total PHA content using fluorescence signals.

#### Equipment and logistics

Equipment will vary based on the protocol, though the fundamentals hold true across all PHA-FQ approaches. The most important considerations center on the fluorochromes: what do they target, how specifically do they bind, what is their fluorescent lifetime, do their Ex/Em wavelengths overlap, etc. Answers to these questions will be context-dependent and will inform which dyes are best. Basic protocols require spectrofluorometers and/or fluorescent microplate readers to measure the Ex/Em spectra of stained cells (69). More advanced methods use flow cytometers and automated staining workflows to increase throughput (64, 70, 71). These advanced methods have achieved runtimes as low as 5–10 minutes while maintaining comparable accuracy (R^2^ = 0.99) to GC-based approaches (63, 64).

#### Pros and cons

Fluorescent methods are quick, safe, simple, and inexpensive compared to other approaches relying on harmful chemicals and complex equipment. Fluorescence microscopy can also be used to verify adequate staining before sample quantitation, making PHA-FQ a useful complement to other approaches. They are also amenable to high-throughput applications (e.g., 96-well microplates or flow cells). Additionally, flow cytometry requires minimal sample volumes up to 100 µL and as low as 10–20 µL (64, 71). However, fluorescent approaches do not always provide the same level of precision that analytical methods like GC or NMR offer, particularly when it comes to assessing individual PHA monomers. This is largely because fluorescent dyes are non-specific and demonstrate variable fluorescence lifetimes (9). Likewise, fluorescent dyes must permeate cell membranes to bind PHA, and permeability may vary across microbes. Future work aimed at developing PHA dyes that are specific to individual PHA monomers (or polymers), maintain steady intensity, and reliably permeate cell membranes would make PHA-FQ an attractive option for rapid quantification that rivals other analytical methods.

Each fluorochrome carries its advantages and disadvantages, which are outlined in detail by Cao et al. (9). For instance, Nile Blue and Nile Red can distinguish between short- and medium-chain length PHAs, yet exhibit off-target binding to lipids in the cell wall (72). This can be mitigated with preliminary experiments that vary the dye concentration, incubation time, pH, temperature, and biomass to determine the optimum conditions for specific binding to PHA while minimizing diffusion into membranes. Researchers must also consider which fluorochrome is ideal given their question(s) of interest, target PHAs, and detection hardware. It is also important to note that the most commonly used fluorochromes have been extensively assessed in pure cultures producing polyhydroxybutyrate (PHB). Further study is needed to understand how reliably other PHAs can be quantified via PHA-FQ in mixed cultures. Even with pure cultures, it is essential to optimize for the given strain and the PHA polymers that those cells produce. Thus, researchers should conduct preliminary optimization experiments when considering a fluorescence-based approach for any strain/fluorochrome pair that has not already been validated.

## SPECIAL CONSIDERATION FOR OTHER FLAVORS OF PHA: OLIGO-PHA AND cPHA

This minireview focuses on methods for detecting and quantifying PHA that serve as carbon and energy reserves for bacteria. This “storage PHA” is made up of high molecular weight (i.e., greater than 10^3^ monomeric residues) polymers that accumulate as intracellular granules. However, PHA takes on other forms with distinct functions. For instance, oligomeric PHAs (oligo-PHA)—which constitute approximately 100 to 200 monomeric units (73)—are found in eukaryotic plasma membranes (74), the calcium-ATPase pump of human erythrocytes (75), the voltage-dependent channel of mitochondria (76), and the porin of *Haemophilus influenzae*’s outer membrane protein P5 (77). Smaller PHA oligomers (less than about 30 units) can also modify proteins via “PHAylation” (73). This so-called “cPHA” covalently binds to peptide residues and plays a crucial role in their function. In one striking example, the TRPM8 channel of mammalian sensory neurons is regulated (78) and post-translationally modified (79) by PHA, thus ensuring its normal function. Oligo-PHA and cPHA also facilitate DNA uptake during transformation (80, 81), exhibit channel activity for inorganic cations (73), mitigate oxidative stress (82–84), and act as a growth-promoting factor in some bacteria (85).

Most researchers use a combination of HPLC and western blot analysis to detect and quantify oligo-PHA and cPHA (86–89). Some have supplemented these approaches with matrix-assisted laser desorption/ionization mass spectrometry to identify specific peptides that are modified with cPHA (79, 89). More recently, researchers applied liquid chromatography time-of-flight spectrometry (90) and Orbitrap LC-MS (91) to analyze the degradation products of poly(3-hydroxybutyrate-co-3-hydroxyhexanoate). Thus, tools are available to investigators interested in pursuing low molecular weight PHAs.

Before detecting, identifying, and/or quantifying oligo-PHA and cPHA, researchers must ensure they are isolating their PHAs of interest from storage PHA. This is straightforward in cases where the organism in question does not produce storage PHA (92); other times, it requires fractionation to separate the intracellular content from membrane-bound oligo-PHA and cPHA (93). In the case of cPHA, researchers must purify the PHA-modified proteins (though they must take care to express PHAylated proteins in organisms with the requisite post-translational machinery) (86, 87, 94). Further complicating this is the conformational mobility of PHA chains—which makes it difficult to analyze PHAylation using cryo-electron microscopy—along with PHA’s chemical instability—which runs afoul of protein preparation protocols for single-crystal X-ray structure determination (94). Thus, researchers interested in exploring the other “flavors” of PHA will face additional technical hurdles that require attention. For a thorough discussion of these PHAs and their scientific history, please see the following articles: (73, 94, 95).

## CONCLUSIONS

**Fig 1:**
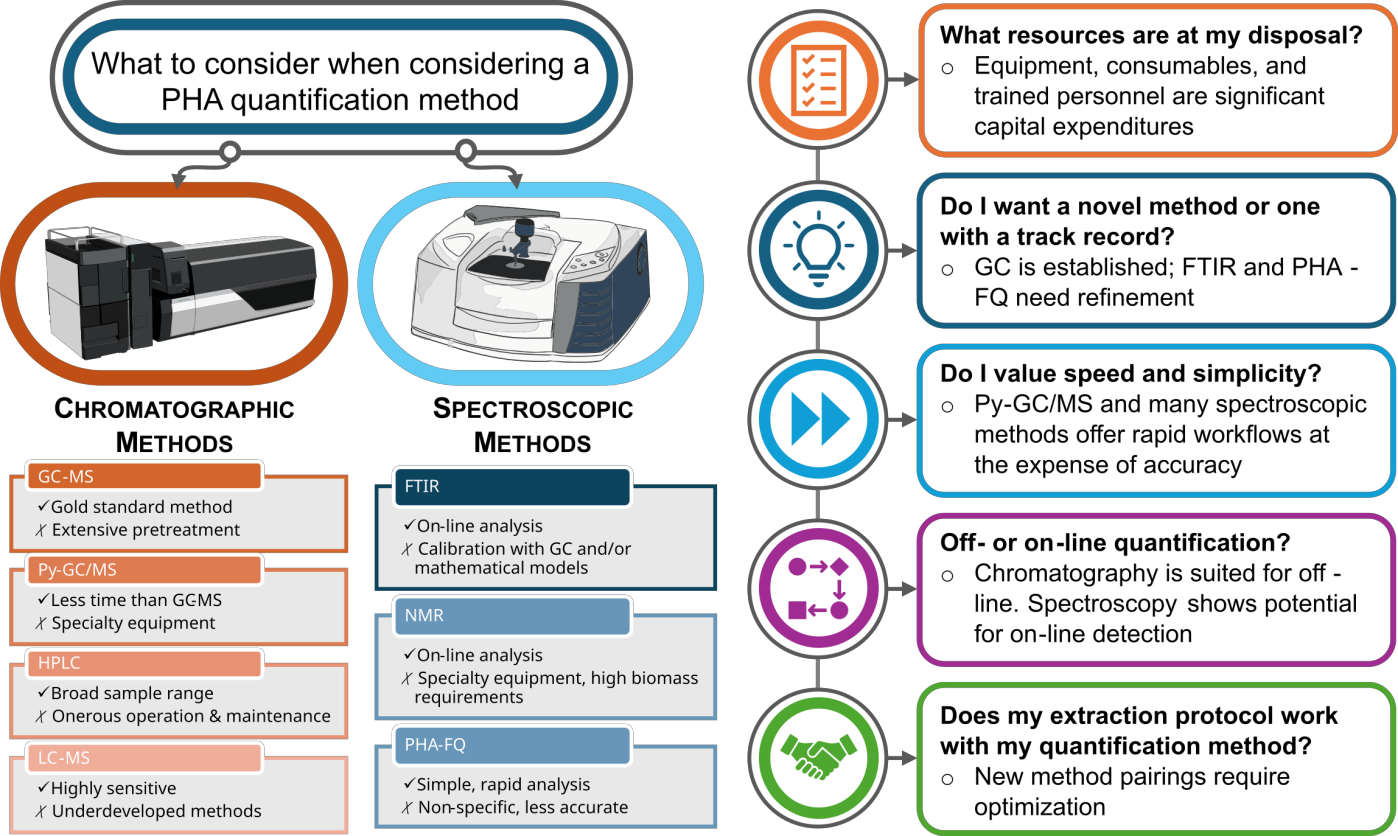
Overview of popular and emerging methods for quantifying microbial polyhydroxyalkanoates discussed in this minireview. The most common and most recently applied methods that provide quantitative information on intracellular PHA content fall into two categories: chromatographic methods and spectroscopic methods. Each method comes with pros and cons, summarized by the check marks and X marks, respectively. On the right, we highlight some questions that researchers should ask themselves when considering methods for quantifying PHA. On-line processes are integrated into a benchtop workflow for real-time processing, dynamic adjustment of parameters in response to changing system properties, and without requiring separate sample collection or processing steps; off-line methods involve multi-step sample collection and processing.

Figure 1 highlights the two categories of methods for quantifying PHA: chromatographic and spectroscopic. The former includes GC-MS, Py-GC/MS, HPLC, and LC-MS; the latter comprises FTIR, NMR, and PHA-FQ. Each approach brings strengths and weaknesses that should be weighed against factors such as equipment, personnel, cost, and experimental design. For example, flow cytometry is the only fluorescence-based technique that can analyze individual cells and assess population homogeneity with respect to PHA accumulation. In contrast, other spectroscopic methods only quantify overall fluorescence intensity, while chromatographic methods are useful for quantifying average PHA content in a sample. However, questions remain about how these factors change for each method above. For instance, what are the limits of detection of each approach, and does that change with different experimental setups? What are the costs in terms of upfront capital expenditures, consumables, and expected maintenance costs? Experimental work and techno-economic analyses can shed more light on these questions. For now, we offer some general guidelines that should help researchers understand the strengths, weaknesses, and use cases for these methods.

### If you value accuracy and sensitivity, consider chromatography; if you value speed and simplicity, select spectroscopy

Spectroscopic methods—particularly FTIR and PHA-FQ—are not as accurate or sensitive as chromatographic methods (96). However, they are much faster, cheaper, and amenable to on-line applications, making them attractive for routine high-throughput analysis. Chromatography is still considered the gold standard, and modern innovations help to mitigate concerns about time demand or hazardous materials. Investigators using chromatography can save time and money by automating parts of the workflow (e.g., by using autosamplers) or, in the case of GC, by incorporating a microfurnace pyrolyzer as part of a Py/GC-MS workflow. Likewise, solvent- or additive-free extraction processes are safe and time-saving alternatives to solvent-based ones.

### If polymer composition matters, consider each method’s limitations

The methods above offer varying degrees of quantitative and qualitative insight. For researchers interested in understanding PHA composition (e.g., heteropolymers and monomers), NMR should be considered the gold standard, as it can differentiate PHA heteropolymers, quantify monomers, and analyze novel functionalized PHAs (96). PHA-FQ can differentiate between short- and medium-chain length PHAs, while FTIR struggles to do so (9, 96). Chromatographic methods vary in their ability to identify individual monomers and distinguish heteropolymer blends, though generally, they effectively separate different monomers (96). The correct balance of qualitative and quantitative insight will be dictated by the research question(s) being pursued.

### If you want to use established workflows, consider Py-GC/MS; if you want to explore emerging approaches, consider LC-MS, FTIR, or PHA-FQ

For those interested in an established, no-fuss, benchmark method, Py/GC-MS is ideal. GC-MS is tried and true in the PHA quantification landscape, and the addition of reactive pyrolysis saves time and money in the long run. On the other hand, researchers seeking to break into the field with *de novo* workflows have opportunities to develop novel methods. FTIR and PHA-FQ are somewhat high-risk investments given the outstanding questions about their accuracy; at the same time, they show tremendous promise as rapid on-line tools for real-time quantification. Similarly, LC-MS is an underexplored approach that is nonetheless viable for labs that have access to the requisite equipment. As these methods continue to be refined, it is possible that they will come to rival the established methods for PHA quantification.
